# You Can’t Think and Hit at the Same Time: Neural Correlates of Baseball Pitch Classification

**DOI:** 10.3389/fnins.2012.00177

**Published:** 2012-12-19

**Authors:** Jason Sherwin, Jordan Muraskin, Paul Sajda

**Affiliations:** ^1^Department of Biomedical Engineering, Columbia UniversityNew York, NY, USA

**Keywords:** single-trial analyses, EEG, perceptual decision-making, baseball, source localization

## Abstract

Hitting a baseball is often described as the most difficult thing to do in sports. A key aptitude of a good hitter is the ability to determine which pitch is coming. This rapid decision requires the batter to make a judgment in a fraction of a second based largely on the trajectory and spin of the ball. When does this decision occur relative to the ball’s trajectory and is it possible to identify neural correlates that represent how the decision evolves over a split second? Using single-trial analysis of electroencephalography (EEG) we address this question within the context of subjects discriminating three types of pitches (fastball, curveball, slider) based on pitch trajectories. We find clear neural signatures of pitch classification and, using signal detection theory, we identify the times of discrimination on a trial-to-trial basis. Based on these neural signatures we estimate neural discrimination distributions as a function of the distance the ball is from the plate. We find all three pitches yield unique distributions, namely the timing of the discriminating neural signatures relative to the position of the ball in its trajectory. For instance, fastballs are discriminated at the earliest points in their trajectory, relative to the two other pitches, which is consistent with the need for some constant time to generate and execute the motor plan for the swing (or inhibition of the swing). We also find incorrect discrimination of a pitch (errors) yields neural sources in Brodmann Area 10, which has been implicated in prospective memory, recall, and task difficulty. In summary, we show that single-trial analysis of EEG yields informative distributions of the relative point in a baseball’s trajectory when the batter makes a decision on which pitch is coming.

## Introduction

The baseball great, Ted Williams, said, “the hardest thing to do in a sport is to hit a baseball.” The hitter has a fraction of a second to decide whether the pitch will be a ball or a strike and whether he will swing or not. Another great, Yogi Berra, summarized the split second timing necessary for this task by saying, “you can’t think and hit at the same time.” Rather, hitters must rely on a rapid decision-making process that tracks the trajectory and speed of the ball with sufficient accuracy to predict its location when it crosses the plate and decide on an appropriate motor response. Due to the different speeds and trajectories that pitches can follow, batters cannot blindly guess and maintain accuracy. A key element of this rapid decision-making process is determining what type of pitch is thrown, e.g., fastball, curveball, or slider, because the type of pitch constrains the possible trajectories of the ball.

Previous studies have examined the pitch classification process using behavioral/physiological markers. For instance, eye movements before and during pitches have been extensively studied (Shank and Haywood, 1987; Kato and Fukuda, 2002; Takeuchi and Inomata, 2009) and have been used to identify optimal visual search strategies employed by expert vs. novice players. These findings show that experts focused their visual (spatial) attention closer to the estimated release point of the pitch, when compared to novices, suggesting that early-trajectory tracking can be crucial for batting performance. The middle phase of the trajectory also has been found to have significant impact on pitch identification. In particular, it has been shown that the middle third of a pitch’s trajectory was most predictive of whether subjects made contact with a pitched softball (De Lucia and Cochran, 1985). This implies that, in addition to early-trajectory tracking, continued tracking of the pitch can influence trajectory estimation.

To date, the only approach directly aimed at investigating neural signatures related to pitch identification has used electroencephalography (EEG) to examine the P300 event-related potential (ERP) in response to two categories of pitches (fastballs and curveballs) when either a correct or incorrect verbal cue preceded the pitch (Radlo et al., 2001). Results showed differences between intermediate and advanced batters in terms of their behavioral accuracy as well as P300 amplitude and latency, with the conclusion being intermediate batters are more easily “fooled” by a preceding cue which was not congruent with the pitch. Though this result suggests how use of prior knowledge and attentional allocation might differ between batters of different aptitudes, it does not directly address the question of what are the neural correlates of the decision event and when they occur relative to the type of pitch thrown and its trajectory in space-time.

In this paper we employ a forced-choice decision-making task, in which subjects must discriminate between three pitch types while under time pressure. We utilize a multivariate classifier to project the neural data (measured via EEG) into a space that optimally separates trials into their predicted pitch class. By doing this on a single-trial basis, we can examine at what point in a pitch’s trajectory the subjects’ underlying neural activity discriminates a given pitch. Furthermore, we utilize source reconstruction techniques (Pascual-Marqui et al., 1999) to identify the neural generators of the decision-making process when the “batter” makes a mistake.

## Materials and Methods

### Subjects

Six subjects participated in the study (one female, mean age-27.33 years). None of the subjects had professional or collegiate baseball experience. All subjects reported normal or corrected vision and no history of neurological problems. Informed consent was obtained from all participants in accordance with the guidelines and approval of the Columbia University Institutional Review Board.

### Stimuli overview and behavioral paradigm

Subjects viewed on a computer monitor 12 blocks of 50 simulated baseball pitches with a mean jittered inter-stimulus interval (ISI) of 2150 ms. The simulated view was that of where the catcher would sit on a standard baseball diamond, i.e., at the end point of the pitch trajectory. From a library of 50 pitches, each coming from one of three pitch types (“fastballs,” “curveballs,” and “sliders”), the subject was presented, on each trial, a pitch chosen at pseudorandom.

For those not familiar with baseball, the three pitches differ in their speed and path through space. A fastball has a trajectory that is straight with very little horizontal or vertical break compared to a simple parabolic trajectory. A curveball has a combination of side spin and top spin that creates both a rightward and downward break. A slider only has sidespin, which creates only a rightward break. Subjects were to discriminate pitches based on these trajectories and the difference in speeds. All pitches were simulated using the equations of motion (see below). Video clips showing one example for each of the three pitch types are provided in the online Supplemental Material.

The subjects were instructed to identify the type of pitch as quickly as possible via a keyboard button response, where each pitch choice was mapped to a unique button (“j,” “k,” and “l”). Subjects were told to respond while the ball was still on the screen. All button responses were executed with the right hand, regardless of handedness. In an initial training phase, subjects learned the general trajectory of each pitch by viewing examples, and for a short practice session they responded with the button response. The practice session contained 20 pitches selected at pseudorandom with no feedback and subjects were asked afterward if they felt comfortable doing the task in the amount of time needed. All subjects responded in the affirmative and the 12 blocks of 50 pitches began with EEG data being recorded. Participants did not receive feedback or collect a reward for their performance; however, they did receive compensation for their time.

A Dell Precision 530 Workstation was used to present the visual stimuli with E-Prime 2.0 (Sharpsburg, PA, USA). The subjects sat in an RF-shielded room 100 cm from the center of the computer screen, where the stimulus display area covered a horizontal angle of ±6.5° and a vertical angle of ±5.0°.

The start of each pitch video clip was the stimulus event by which EEG time-locking occurred. Stimulus events were passed to the EEG recording system through a TTL pulse in the event channel. In *post hoc* analysis, response events were synchronized to the EEG via their latencies from the stimulus event.

### Pitch simulations

Each pitch video clip was created using a differential equation solver in Matlab 2010a (Mathworks, Natick, MA, USA; see Pitch Simulations below) and exported to an audio-video Interleaved (.avi) movie file (see Supplementary Material for examples) sampled at 60 Hz (refresh rate of display monitor). Most baseball pitches can be simulated using six-coupled differential equations (Armenti, 1992; Adair, 1995).

$$\begin{array}{llll} \frac{dx}{dt} & =v_{x} & & \text{(1)}\\ \frac{dy}{dt} & =v_{y} & & \text{(2)}\\ \frac{dy}{dt} & =v_{z} & & \text{(3)}\\ \frac{dv_{x}}{dt} & =-F\left(v\right)vv_{x}+B\omega \left(v_{z}\sin\phi -v_{y}\cos\phi \right) & & \text{(4)}\\ \frac{dv_{y}}{dt} & =-F\left(v\right)vv_{y}+B\omega v_{x}\cos\phi & & \text{(5)}\\ \frac{dv_{z}}{dt} & =-g-F\left(v\right)vv_{z}-B\omega v_{x}\sin\phi & & \text{(6)}\\ F\left(v\right) & =0.0039+\frac{0.0058}{1+e^{\left(v-v_{d}\right)∕\Delta }} & & \text{(7)} \end{array}$$

The first three equations specify the change in spatial location in each direction, which equals the velocity of the baseball. The last three equations specify the accelerations due to the drag [*F*(*v*)], the Magnus force (*B*), and gravity (*g*) acting on the baseball. Equation 7 is used to calculate the drag force at different velocities with *v_d_* = 35 m/s and Δ = 5 m/s. The Magnus force (*B*), which occurs due to differential drag on a spinning object, is approximated here to be 4.1 × 10^−4^ (dimensionless). After specifying the initial conditions [*x*_0_, *y*_0_, *z*_0_, *v_x_0*, *v_y_0*, *v_z_0*, ω (rotational frequency)], the six ordinary differential equations were solved in MATLAB.

The three pitches – fastball, curveball, and slider – have well-defined individual initial conditions. To create each pitch, we only need to vary the initial velocity and the rotation angle. For each pitch class, 50 pitches were created by randomly sampling distributions of initial conditions for velocity, rotation angle, launch angle, and horizontal launch angle. The values and distributions used for each pitch class are specified in Table 1.

**Table 1:** **Parameters for generating pitch trajectories**.

	Initial velocity (MPH)	Rotation angle (°)	Vertical launch angle (°)	Horizontal launch angle (°)	Rotational frequency (rpm)	Duration (s)
Fastball	83 ± 3	270 ± 3	0.5 ± 0.3	0.7 ± 0.3	1800	0.53 ± 0.01
Curveball	70 ± 3	50 ± 10	1.7 ± 3	0.7 ± 0.3	1800	0.64 ± 0.02
Slider	75 ± 3	0 ± 5	1.7 ± 3	0.7 ± 0.3	1800	0.59 ± 0.02

For each simulated pitch, a blue circle was plotted on a gray grid for every frame of the trajectory. The size of the blue circle increased as it approached the viewer, so as to give the illusion of depth. When the ball crossed “home plate,” the blue circle disappeared. The frames were compressed into .avi movie format (see examples of each pitch simulation in Supplementary Material). The trajectories, for each simulation, were saved in a separate file for later use.

### Data acquisition

Electroencephalography data was acquired in an electrostatically shielded room (ETS-Lindgren, Glendale Heights, IL, USA) using a BioSemi Active Two AD Box ADC-12 (BioSemi, The Netherlands) amplifier from 64 scalp electrodes. Data were sampled at 2048 Hz. A software-based 0.5 Hz high pass filter was used to remove DC drifts and 60 and 120 Hz (harmonic) notch filters were applied to minimize line noise artifacts. These filters were designed to be linear-phase to minimize delay distortions. Stimulus events – i.e., pitch-movie start times and pitch types – were recorded on separate channels.

Independent components analysis (ICA) was run using EEGLAB (Delorme and Makeig, 2004) to remove eye-blink artifacts. In stimulus-locked epoching (−1000 to 1500 ms), the average baseline was removed using data from −1000 to 0 ms. After epoching to stimulus events, an automatic artifact epoch rejection algorithm from EEGLAB (Delorme and Makeig, 2004) was run to remove all epochs that exceeded a probability threshold of 5 SDs from the average. Similarly, in response-locked epoching (−1500 to 1000 ms), the average baseline was removed from −1500 to −500 ms and the same automatic artifact epoch rejection algorithm was run.

### Data analysis

We performed a single-trial analysis of the filtered, epoched, and artifact-removed EEG to discriminate between a set of stimulus or response conditions. First, we considered only behaviorally correct pitches, where the user’s response was within 100 ms of the end of the pitches’ trajectory, and trained the classifier to classify a given pitch (e.g., a fastball) vs. pitches of the other classes (e.g., curveball and slider). Second, we classified behaviorally correct vs. incorrect pitches within each pitch class (e.g., correctly identified fastballs vs. incorrectly identified fastballs). A table summarizing the classification analysis is shown in Table 2.

**Table 2:** **Definition of classes used in discrimination analysis**.

Correct pitches		Correct-incorrect	
Class 1	Class 2	Class 1	Class 2
Fastball	Not-fastball	Correct fastball	Incorrect fastball
Curveball	Not-curveball	Correct curveball	Incorrect curveball
Slider	Not-slider	Correct slider	Incorrect slider

Logistic regression was used as a classifier to find an optimal projection for discriminating between the chosen two conditions over a specific temporal window (Parra et al., 2002, 2005; Conroy and Sajda, 2012). This approach has been previously applied to identify neural components underlying rapid perceptual decision-making (Gerson et al., 2005; Philiastides and Sajda, 2006; Philiastides et al., 2006). Specifically, we defined a training window starting at either a pre-stimulus or post-stimulus onset time τ, with a duration of δ, and used logistic regression to estimate a spatial weighting vector ${\vec{w}}_{\tau ,\delta }^{T}$ which maximally discriminates between EEG sensor array signals *X* for each class (e.g., fastballs vs. not-fastballs):

(8) $$\vec{y}={\vec{w}}_{\tau ,\delta }^{T}X$$

In Eq. 8, *X* is an *N *× *T* matrix (*N* sensors and *T* time samples). The result is a “discriminating component” $\vec{y}$ that is specific to activity correlated with each condition, while minimizing activity correlated with both task conditions. The term “component” is used instead of “source” to make it clear that this is a projection of all activity correlated with the underlying source. For our experiments, the duration of the training window (δ) was 50 ms and the center the window (τ) was varied across time τ = 0, 25, 50,…, 975, 1000 ms in 25 ms steps for stimulus-locked, and was varied across time τ=-575,-550,…,550, 575 ms in 25 ms steps for response-locked. We used the re-weighted least squares algorithm to learn the optimal discriminating spatial weighting vector ${\vec{w}}_{\tau ,\delta }^{T}$ (Jordan and Jacobs, 1994).

In order to provide a functional neuroanatomical interpretation of the resultant discriminating activity, and due to the linearity of the model, we computed the electrical coupling coefficients (Eq. 9).

(9) $$\vec{a}=\frac{X\vec{y}}{\vec{y}∙\vec{y}}$$

This equation describes the electrical coupling $\vec{a}$ of the discriminating component $\vec{y}$ that explains most of the activity *X*.

We quantified the performance of the linear discriminator by the area under the receiver operator characteristic (ROC) curve, referred to here as *A_z_*, using a leave-one-out procedure (Duda, 2001). We used the ROC *A_z_* metric to characterize the discrimination performance as a function of sliding our training window from 0 ms pre-stimulus to 1000 ms post-stimulus (i.e., varying τ) for stimulus-locked and −575 ms pre-response to 575 ms post-response for response-locked. These time periods provided substantial time both after the stimulus and behavioral response (button press) to observe any electrophysiological response to the pitch.

We quantified the statistical significance of *A_z_* in each window (τ) using a relabeling procedure. With 41 windows for stimulus-locked and 47 for response-locked, we had to correct for this number of comparisons within stimulus- and response-locked leave-one-out respectively. To have a Bonferroni corrected *p* < 0.05 threshold in both locking conditions, we ran enough permutations to have a suitable number of samples within the *p* < 0.001 threshold (i.e., *p* < 0.05/41 and *p* < 0.05/47). To this end, we randomized the truth labels (i.e., pitch was a fastball, curveball, or slider) for each trial and retrained the classifier. This was done 3750 times for each subject (1250 permutations for each pitch comparison combination), giving a total of 22,500 permutations for a group level analysis. The *A_z_* values from these permutations were used to establish a *p* < 0.001 threshold, i.e., a *p* < 0.05 Bonferroni corrected significance level. All significant results are thus reported at *p* < 0.05 corrected for multiple comparisons.

### Component-informed source localization

**Figure 1:**
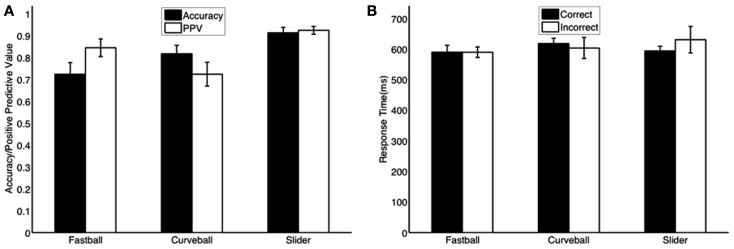
**Mean behavioral responses across subjects for (A) accuracy and positive predictive value (PPV) and (B) mean response times for correctly and incorrectly identified pitches**. All bars are plotted with standard errors (*N* = 6).

We used source localization to investigate the differences between correctly vs. incorrectly identified pitches. First, we classified the stimulus-locked EEG data of incorrectly vs. correctly identified pitches, as summarized in the right half of Figure 1 and Table 2. This was done on a subject-specific basis, except for one subject for whom there were no errors in discriminating the slider; therefore that subject was removed so as not to bias the results. For each of the remaining five subjects, we then selected the window at which the LOO *A_z_* value was maximum, with the constraint that the subject-specific maximum was not outside the range of 3 SEs of the pitch-specific mean peak timing. This was done to ensure that the localization analysis was investigating a temporally common phenomenon across subjects.

Using these markers in time, we trial-averaged the EEG sensor data across all epochs that were either correctly identified or incorrectly identified, creating a grand average ERP for each of the five subjects, for each pitch, and for both accuracies. Given five subjects, three pitches, and two conditions, this results in a total of 15 ERPs for each condition (i.e., for correctly identified and incorrectly identified pitches).

Using these grand average ERP values, we then utilized a source localization algorithm (sLoreta; Pascual-Marqui et al., 1999) to estimate the most likely cortical source distributions. This algorithm solves for the most likely current source distribution in the cortex based on EEG sensor data and array topology. We used these distributions to compare the incorrect vs. correct classification conditions across subjects and pitches.

## Results

### Behavioral performance

From the behavioral data summarized in Figure 1, we see mean accuracy was 72, 82, and 91% for fastballs, curveballs, and sliders, respectively. We also calculated the positive predictive value (PPV) – i.e., number of true positives divided by the sum of true positives and false positives-for each pitch class. The PPV of each pitch class showed that the subjects were confident when selecting sliders and fastballs, however, for curveballs the PPV is significantly less than the accuracy, indicating that the curveball could possibly be the default choice for the subjects – i.e., it was often selected as a false positive.

**Figure 2:**
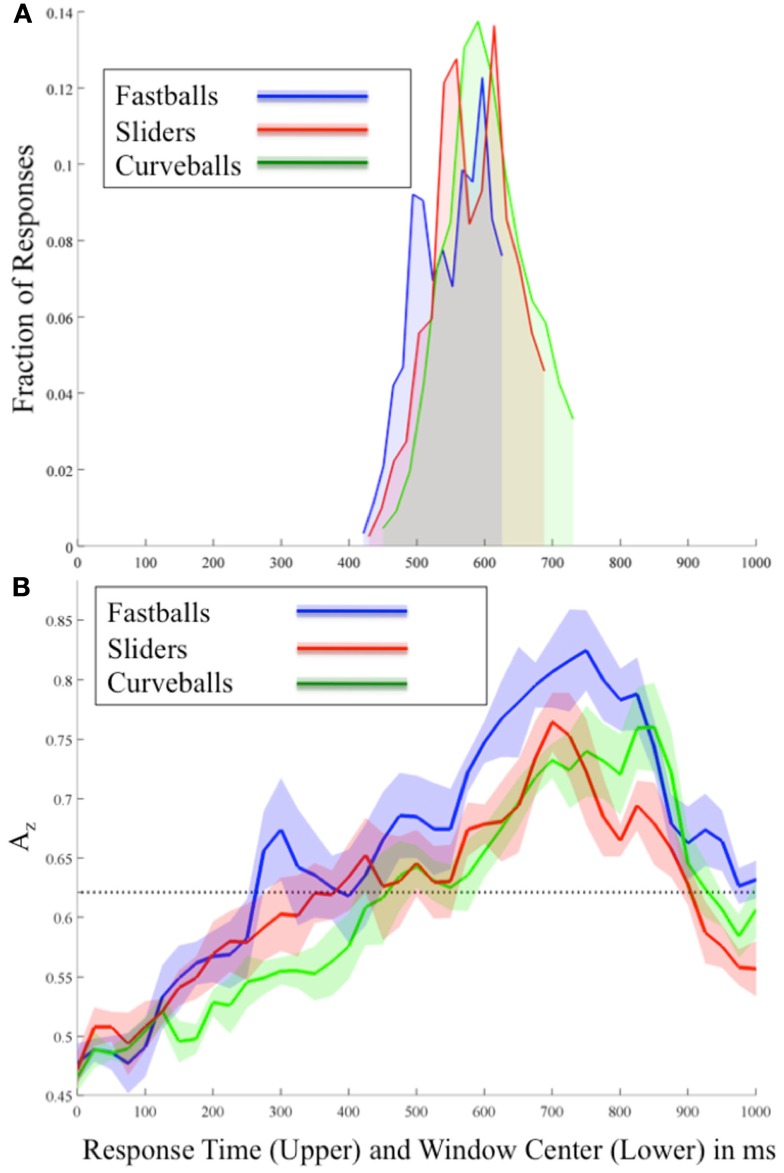
**(A)** Behavioral and **(B)** stimulus-locked EEG discrimination results for each pitch averaged across all subjects. In **(B)** each *A_z_* curve shows the mean and standard error bands computed using leave-one-out discrimination for the indicated pitch vs. other pitches (e.g., for fastballs, the discrimination is between fastballs and not-fastballs; i.e., curveballs and sliders). The significance line (dotted) is corrected for multiple comparisons (line at *p* = 0.05 Bonferroni corrected for 41 time window comparisons). Note the two plots are time aligned and have the same time-scale so as to compare the timing of EEG discrimination and behavioral response.

The behavioral results (Figure 2A) show response times as a probability density function with truncations on the right-side of the distributions, indicating the threshold enforced 100 ms after the pitch arrived at the plate. Mean response times for correctly identified pitches were 590, 618, and 594 ms for fastballs, curveballs, and sliders, respectively. The first peaks of each pitch’s response distribution are at 494 ms (fastballs), 558 ms (sliders), and 590 ms (curveballs).

To test whether response times were significantly different from one other, we ran a three-way ANOVA with the three factors being subject, pitch type, and correct/incorrect classification. The subject factor was treated as a random effect while the other two factors were treated as fixed effects in the model. We found no significant differences in all of the comparisons tested (*p* > 0.05)-difference between pitch types (*p* = 0.08, *F* = 3.38, *df* = 2), difference between correct/incorrect (*p* = 0.68, *F* = 10.86, *df* = 5), the interaction between pitch type and correct/incorrect (*p* = 0.24, *F* = 1.66, *df* = 10). The ANOVA indicates that the mean behavioral responses are not statistically different with regards to the pitch type or whether the subject classified the pitch correctly.

### Neural markers of correctly identified pitches: Stimulus-locked analysis

Figure 2B shows the mean discrimination performance (*A_z_* values) across all subjects and for each pitch using stimulus-locked EEG discrimination. From this stimulus-locked analysis, we see a relationship between the speed of the pitch and the timing of peaks in both neural and behavioral data. In particular, in Figure 2B correctly identified fastballs exhibit the earliest significant EEG discrimination (300 ms), while sliders (425 ms), and curveballs (500 ms) follow. As expected, the sequence of these peaks follows the relative speeds of these pitches, i.e., fastballs, sliders, and then curveballs. Comparing these peaks to the behavioral results of Figure 2A, each of the response distribution peaks immediately follows the relative timing of each pitch’s first significant neural discrimination.

As the response times show, the stimulus-locked discrimination overlaps the responses ∼420–720 ms, so it is difficult to isolate non-motor elements in the signal during these time periods. However, after the responses, the highest peaks of discrimination for each pitch are seen (750 ms for fastballs, 700 ms for sliders, and 850 ms for curveballs). To test whether these post-response peaks are due to the differences in the response time distributions, a separate analysis was run on a subset of the data where the RT distributions were matched between classes. These large peaks remain and are therefore possibly indicating a post-response evaluative process specific to identifying each pitch correctly.

**Figure 3:**
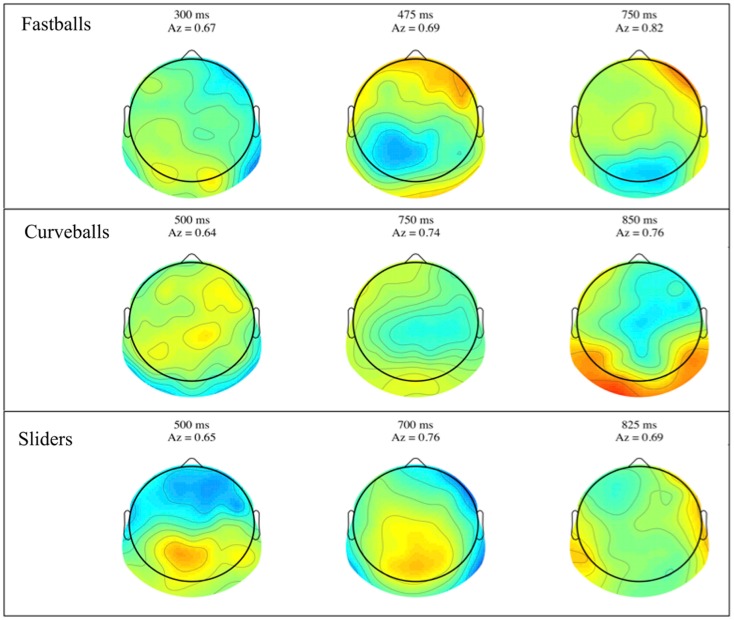
**Group averaged stimulus-locked forward models, shown as scalp maps, for each of the three pitch types**. Only behaviorally correct trials are used to estimate these forward models. For each pitch, three time windows are shown for discriminating components estimated for those windows. The center time of each window is given above each plot together with that window’s discrimination (*A_z_*) value.

Group mean stimulus-locked forward models, shown as scalp plots, are given in Figure 3. Plots are for selected time points across all three pitches, where the center of the discrimination window is indicated at the top of each subfigure. Dark red and blue colors indicate strong discriminatory power from electrodes in that region. While the areas of discrimination change over time for each pitch type, discrimination power is consistently located in the posterior and occipital portions of the scalp plots.

### Neural markers of correctly identified pitches: Response-locked analysis

**Figure 4:**
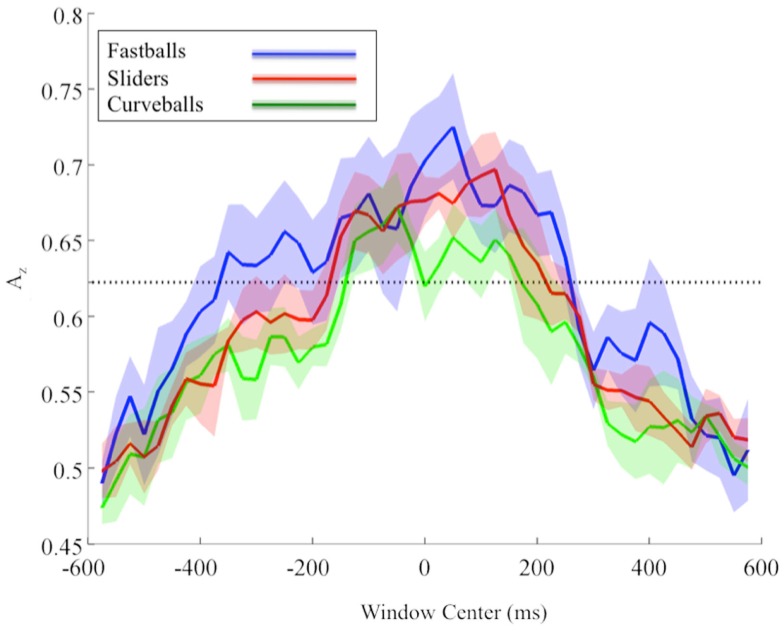
**Group mean and standard error bands for response-locked leave-one-out EEG discrimination performance across all subjects**. The significance line (dotted) is corrected for multiple comparisons (line at *p* = 0.05, Bonferroni corrected for 47 time window comparisons).

Due to the possibility of a motor confound in the neural signal, we also classified EEG data locked to the response times (see Figure 4). Once again, using only correctly identified pitches, we calculated the *A_z_* values across all subjects and for each pitch.

Similar to the stimulus-locked results, we find significant pre-response peaks (Bonferroni corrected, *p* = 0.05) for each pitch that follow the relative speeds of each pitch. In particular, the mean pre-response peak for fastballs (−350 ms), precedes that of sliders (−125 ms), and then curveballs (−50 ms). As with the stimulus-locked discrimination, there is a post-response period in which the mean discrimination is as high or higher than it was pre-response for each pitch (fastballs at +50 ms, sliders at +125 ms, and curveballs at +50 and +125 ms), indicating a possible post-response evaluation of the evidence gathering and subsequent decision.

**Figure 5:**
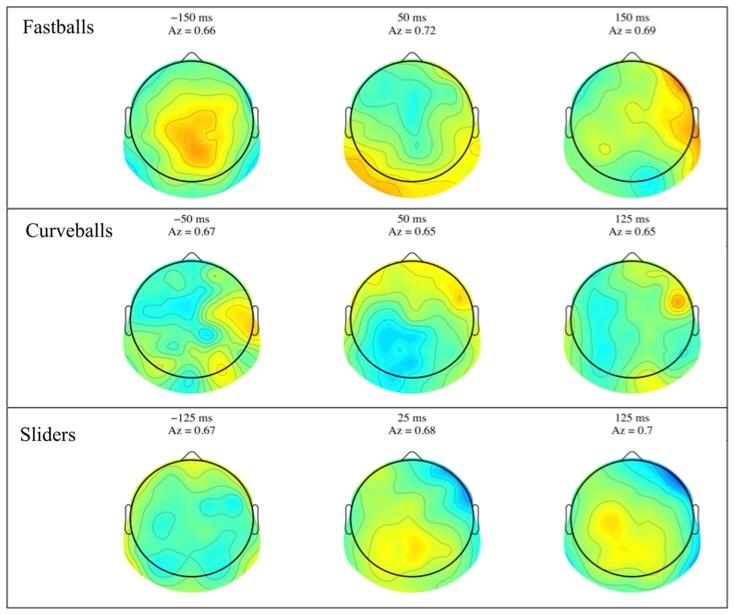
**Group averaged response-locked forward models, shown as scalp maps, for each of the three pitch types**. Only behaviorally correct trials are used to estimate these forward models. For each pitch, three time windows are shown for discriminating components estimated for those windows. The center time of each window is given above each plot together with that window’s discrimination (*A_z_*) value.

Similar to the stimulus-locked figure, mean response-locked forward model scalp plots are shown in Figure 5. Again, we can see that discrimination power is located in the posterior of the brain and the spatial distributions change over time. Only the slider post-response scalp map shows a pattern that might be indicative of a button response (125 ms window shows lateralized contralateral discriminatory activity – i.e., left side activity indicative of a right handed button press).

### Distribution across pitch trajectory of maximally discriminating neural components

**Figure 6:**
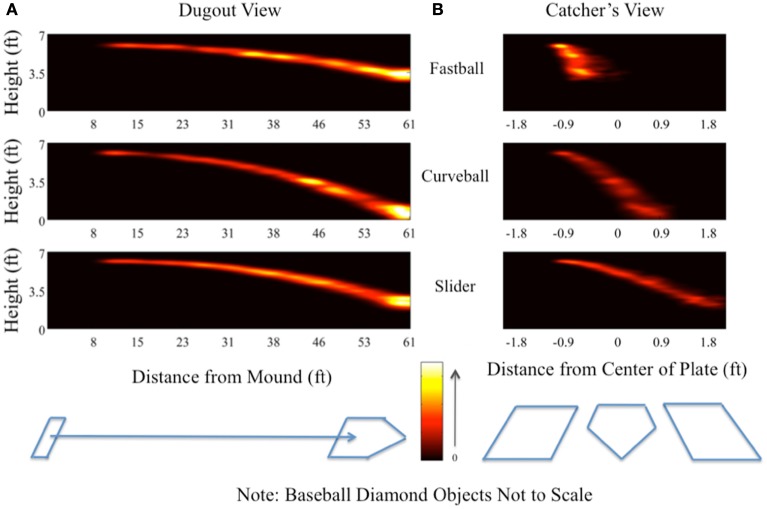
**“Dugout” (A) and “catcher’s” (B) views showing spatial distribution of single-trial peak discrimination across all subjects**. For each subject and for each trial, the timing of peak significant discrimination (i.e., maximum value of *A_z_* above the *p* = 0.05 Bonferroni corrected threshold) from the leave-one-out analysis was related to the trajectory of the pitch on a given trial through the differential equation used to calculate its motion from the simulated pitcher’s release [0 ft on the *x*-axis of **(A)**] to construct a marker in space. Repeating this process for all subjects and all trials results in a distribution of points in space for each pitch at which peak discrimination occurred in a given trial and for a given pitch. The “dugout” view **(A)** shows a two-dimensional projection along the initial trajectory of the pitch, while the “catcher’s” view **(B)** shows the two-dimensional projection in the plane perpendicular to the pitch’s initial trajectory. Color heat maps indicate the height of the distribution.

Using our stimulus-locked single-trial analysis, we constructed spatial distributions of the neural markers across trials and compared these distributions across pitch types. Specifically, we computed the spatial position of the maximum *y* value for τ ∈ ℛ |0< x < τ_plate_), where τ_plate_ is the time at which the pitch reaches the end of its trajectory, i.e., at home plate. Doing this analysis across all subjects, and for each pitch, we created a “heat map” representation of the probability density function of the spatial position in the pitch trajectory having the most discriminating neural component. Figure 6 shows these distributions for both a side view of the trajectory (i.e., the “dugout view”) and a heads-on view (i.e., the “catcher’s view”). Features of the baseball diamond, such as the pitcher’s rubber, home plate, and the batter’s boxes, have been added (not to scale) for a frame of reference.

Both views provide insight into the amount of evidence (e.g., time integration and spatial information) required to classify each pitch. From Figure 6A, we see that common to all pitches is peak discrimination happening when the ball arrives at the plate, likely a result of the tight coupling of the decision with the motor response. However there are significant differences between these distributions if we consider the probabilities prior to when the ball reaches the plate. For example, the fastball has discrimination peaks from mid-trajectory to home plate. The slider also shows this trend, though the probability mass is spread more throughout the trajectory and is thus less localized in terms of pitch position. Finally, the curveball (i.e., the slowest pitch) shows local peaks in the spatial distribution in the later half of the trajectory, presumably due to the slower speed of this pitch relative to the fastball and slider.

Similarly, the catcher’s view (Figure 6B) shows peak discrimination early in the fastball’s trajectory, whereas both the slider and the curveball exhibit distributions spread across the entire trajectory of the pitch. Together, these plots indicate that, due to the higher relative speed and distinct trajectory of the fastball compared to the curveball and slider, the decision process resulting in a correct identification of the fastball occurs earlier in the spatial trajectory than it does for the sliders and curveballs, both of which take incrementally longer periods of time to arrive at the plate.

### Source localization of incorrectly identified pitches

Thus far, our results have focused on an analysis of those pitches (trials) that are correctly classified by the subject(s). However as Figure 1 shows, not all pitches were classified correctly. We therefore analyzed correct vs. incorrect pitch classifications in terms of the EEG discriminating components. Using the temporal windows having maximum *A_z_* within each subject and pitch combination (see Materials and Methods), we found pitch-specific common windows covering 570 ± 89, 744 ± 69, and 522 ± 96 ms for fastballs, curveballs, and sliders, respectively. For only 2 of the 15 combinations of three pitches and five subjects, we found maximum *A_z_* values that exceeded 3 SEs from these mean timings. For these two cases, we chose the second maximum peak in *A_z_* from the next concave down region in the epoch. These points turned out to be within 3 SEs of mean peak Az timings. Thus all analysis was in a temporal period that could be considered as “common decision processing” at a group level.

**Figure 7:**
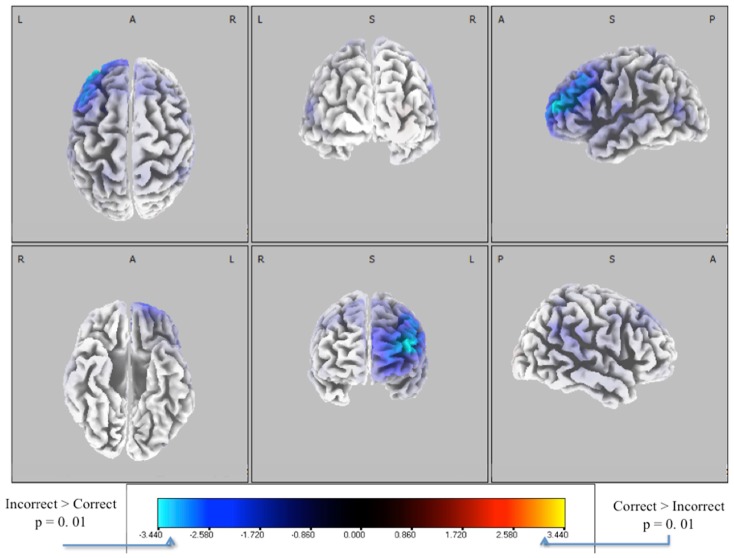
**Six-views of neuronal current independent groups *t*-tests (in corrects in purple/blue, corrects in orange/yellow) for all pitches combined**. In both plots, the log of the *F*-ratio for each voxel is shown [*F*(1, 13)] with brighter colors indicating higher values of the *t*-statistic according to the color scale. Significance was established with a permutation test (5000 permutations). The EEG data used for these neuronal source calculations were the result of averaging scalp potentials at each channel at the subject-specific peak discrimination during the group average discrimination peaks. This analysis is done for each subject (one subject removed due to no errors in sliders), for both correctly and incorrectly identified pitches, and for each pitch type.

We extracted EEG data from these windows and solved for the source distributions using sLoreta (see Materials and Methods). We did a paired *t*-test for correct vs. incorrect identification distributions, with the resulting *t*-distribution of the log of the *F*-ratio [*F*(1, 13)] shown in Figure 7. Five thousand permutations were used to establish significance levels (*p* < 0.01) for the null hypothesis of no difference in activity between incorrectly identified and correctly identified pitches. Though the hypothesis of correctly identified pitches shows no significant similarities (red), that of incorrectly identified pitches (blue) shows a common neuronal current source located in the left frontal cortex, showing peaks in Brodmann Area 10 [BA 10, MNI (−35, 55, 20)]. This result indicates left-lateralized common neuronal activity when subjects incorrectly identify the pitch, i.e., when they “miss.” This result is invariant to the type of pitch, given that all pitches were considered in this analysis.

## Discussion

In this paper, we have shown that we are able to identify neural correlates, in scalp EEG, of baseball pitch classification. Furthermore, using single-trial analysis, we identified at what points in the pitches’ trajectory the EEG correlate is most discriminating, enabling us to infer how decision points may vary across trials and pitch types. Finally, we also showed that there appears to be a common neural generator when subjects make decision errors in this task. We now consider these results in the context of previous literature.

### Related behavioral studies

Though the paradigms and data collection methods are slightly different, we can view our results in the context of other baseball experiments in which behavioral markers were used as performance indicators. In particular, Takeuchi and Inomata (2009) and Kato and Fukuda (2002) used behavioral responses and eye-tracking to monitor subject performance in judging balls and strikes between experts and novices. While the task is slightly different than ours (i.e., balls and strikes vs. fastballs, curveballs, and sliders), and our paradigm did not test experts vs. novices, the fundamental concept of a forced-choice decision based on trajectory remains the same.

The key point from both of these studies is that the expert group’s better performance depends on early-trajectory tracking of the ball from the release point, when compared to that of novices. We see some concordance between this result and our neural discrimination results. Recalling that these studies only focused on fastballs, we can compare this early-trajectory preference for experts to the neural discrimination for correct pitches shown in Figure 2. Being the fastest of the three pitches in our paradigm, subjects had a higher probability of maximum discrimination early in the fastball trajectory when compared to the curveball, with differences with the slider not being nearly as significant. This is indicated by the brighter intensity of the top row’s heat map in Figure 6 on early parts of the fastball pitch’s trajectory, when compared to that of the other pitches. Though while we find that the early parts of the trajectory are less likely, on a single-trial basis, for discriminating neural activity, we do find that most trials are discriminable from the EEG in the middle and latter periods of the pitch trajectory.

De Lucia and Cochran (1985) conducted a behavioral study with findings supporting the importance of the latter parts of the pitch trajectory (middle and late phases). In this study, each third of a fast-pitch softball pitch was visually masked and the subjects’ ability to make contact was used as a behavioral metric on performance. While the masking of each third of the trajectory dropped performance from the non-masking condition, the masking of the middle third proved to have the most significant impact on performance. Once again, using the fast-pitch softball equivalent of an overhand fastball, we can compare this result to the top row of Figure 6 and see that their result is in general concordance with our neural discrimination results. In particular, we see a peak in discrimination in the middle third between 32 and 40 ft from the pitcher’s release. But as with the eye-tracking result, this behavioral-only result does not tell the complete story. Rather, for the latter part of the trajectory the EEG at those time/positions contains substantial discriminatory information for correct fastballs, as indicated by the brightest peaks close to the plate in the top row of Figure 6. Our results suggest that single-trial discrimination of neural markers of pitch classification provides additional insight into the timing of decision-making processes of the “hitter,” particularly with respect to how these may vary across pitch and trial and both confirms and complements previous results using only eye-tracking and behavioral responses.

### Comparison to other results relating neural measures to baseball pitch classification

As mentioned in the Introduction, we are aware of only one other baseball experiment with neural data. In that experiment, Radlo et al. (2001) measured the P300 of subjects in a cued vs. non-cued condition for fastballs and curveballs. To directly compare this study to ours, we consider only the non-cued condition since we provide no evidence to the subject before the ball appears onscreen from the right handed pitcher’s release point. The response distributions for both pitches were faster for the Radlo study than they were for ours. However, we believe that this is due to their experiment being only two-choice, whereas ours is three-choice. Furthermore, the difference between a fastball and a curveball are, trajectory-wise, quite large, whereas the difference between the two breaking balls used in our task (curveball and slider) is smaller. Considering both of these differences in paradigm structure, we expect that the response time distributions of Radlo et al. would be shifted to earlier times relative to ours, even though the pitch speeds are approximately the same.

Using the P300 as an indicator of pitch-specific neural response in the subjects, we find concordance between the sequencing of our pre-response peaks of leave-one-out discrimination across subjects with their results. Radlo et al. (2001) found that fastball P300 peaks preceded those of curveballs and we found this relative sequencing with our discriminator for both stimulus-locked (Figure 2B) and response-locked (Figure 4) analysis. Due to the earlier response times of their subject population though, it is difficult to compare the exact timings of their results and ours.

Lastly, Radlo et al. (2001) used an experimental paradigm which was designed to evoke a P300 (i.e., they used an oddball task, where the oddball was defined by the incorrect cue being given only 25% of the time) whereas our task is a three choice task in which all three pitch types are equally likely. Thus we would not expect our results to generate a typical P300 neural response. However, recalling that their P300 peak latencies followed their response time distributions, whereas our discriminating component preceded responses in both stimulus- and response-locked analyses, we can reason that our discriminator is utilizing a neural signal that precedes the response, and therefore is not the same signal that Radlo et al. find on a group level.

### Incorrect decisions and prefrontal cortices

The other major finding of this paper is that for incorrectly identified pitches there is a common neuronal current source active across our population. The timing for this activity is determined from the peak EEG-based discrimination within each pitch and within each subject; therefore it is controlled for the variability between both subjects and pitches. The common neuronal activity is found from the stimulus-locked discrimination between correct and incorrect identifications. The timings of these peaks are after nearly all of the response time distribution. So while likely not an indicator of upcoming performance, this common neuronal current source, which is peaked at MNI (−35, 55, 20) in BA 10, could be a largely *post hoc* evaluation of the executed decision process.

While direct connection of this area in a baseball task has not been reported in the literature, other studies have found its role in prospective and working memory. For instance in PET studies, Burgess et al. (2001, 2003) and Okuda et al. (2007) found activation of this area in a prospective memory task, i.e., a task to be executed after a period of delay. fMRI studies have also shown this area to be active for working memory and other recall-based tasks (Schacter et al., 1996; Gilbert et al., 2006). In light of these findings, it is possible that the activation we find is a *post hoc* evaluation of the errant decision process, since such a process must engage the prospective and working memory regions of the prefrontal cortex.

Furthermore, this area has also been implicated in task difficulty. For example, Mangina et al. (2009) find that this location in BA 10, among others, plays a role in monitoring task difficulty. Considering our result from incorrect trials in this context, it is possible that on these trials, the subjects simply could not integrate the spatio-temporal information fast enough to make a correct decision, thereby causing an incorrect response, which is only realized in the post-response self-evaluation period. Even though this aspect of the task was not the primary focus of our study, since no explicit feedback was given to the subject on whether their response was correct, it is nonetheless a possible reason for the neuronal current source localizing in BA 10.

## Conclusion

In summary we have identified neural markers that can be used to determine when/where on the pitch trajectory the “hitter” integrates significant evidence for classifying the pitch. We also identified BA10 as an activated region during incorrect trials. Future work will focus on using combined EEG and fMRI (Goldman et al., 2009) to localize, with better spatial resolution, the brain areas involved in classifying pitches. Another possible research question that results from this work is “how do expert batters compare to novices in terms of their corresponding neural markers of pitch classification?” Studying professional or semi-professional hitters may give us insight into performance monitoring and more efficient strategies in identifying pitches. Finally, and more generally, our approach can be extended to other physics-based models of visual stimuli. Interesting to consider would be whether EEG components would be useful for identifying specific physical parameters to which subjects are particularly sensitive.

## Conflict of Interest Statement

The authors declare that the research was conducted in the absence of any commercial or financial relationships that could be construed as a potential conflict of interest.

## Supplementary Material

The Supplementary Material for this article can be found online at http://www.frontiersin.org/Decision_Neuroscience/10.3389/fnins.2012.00177/abstract
